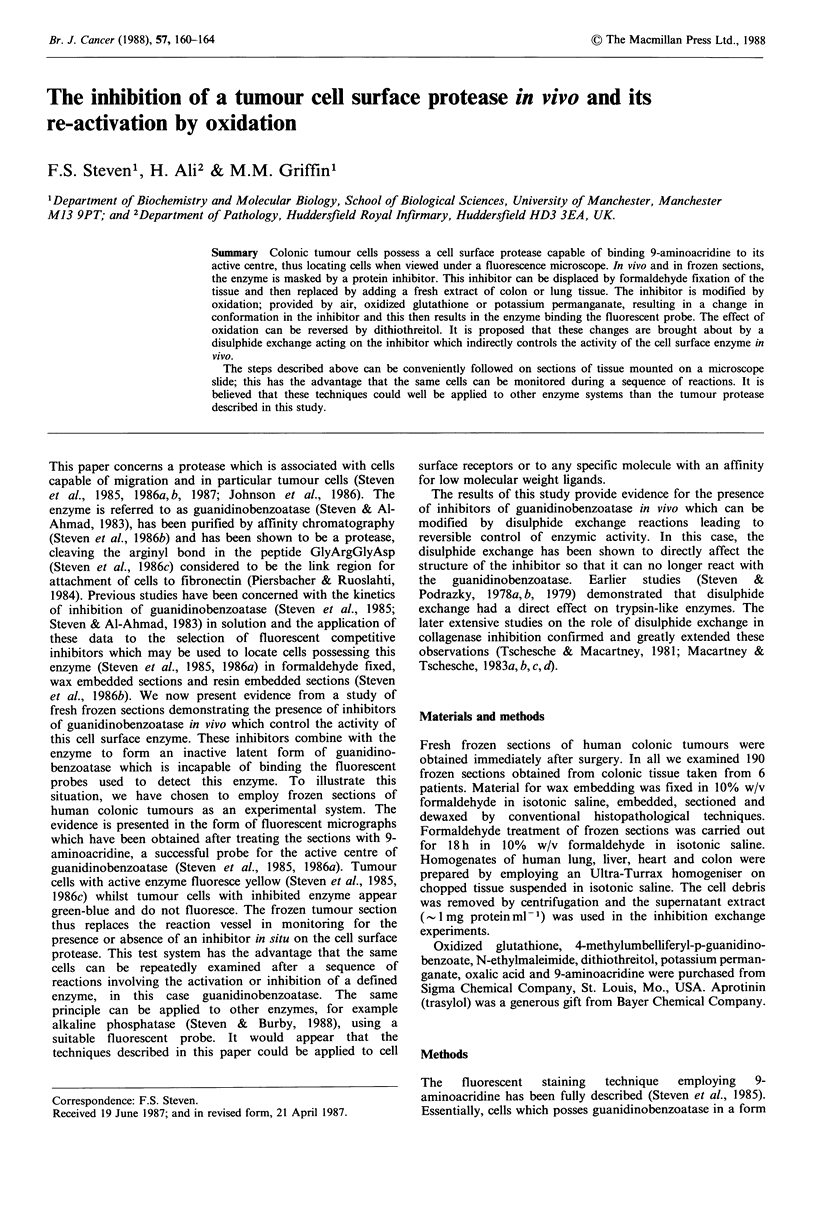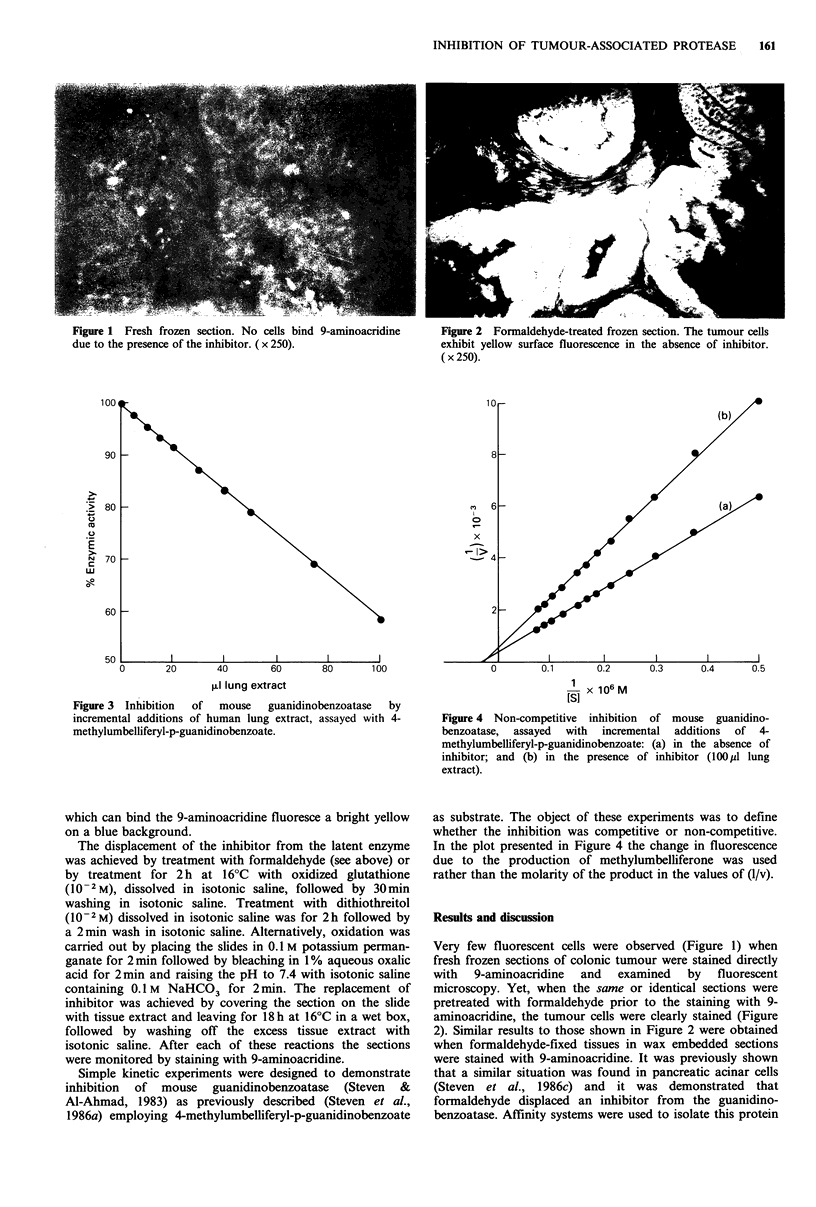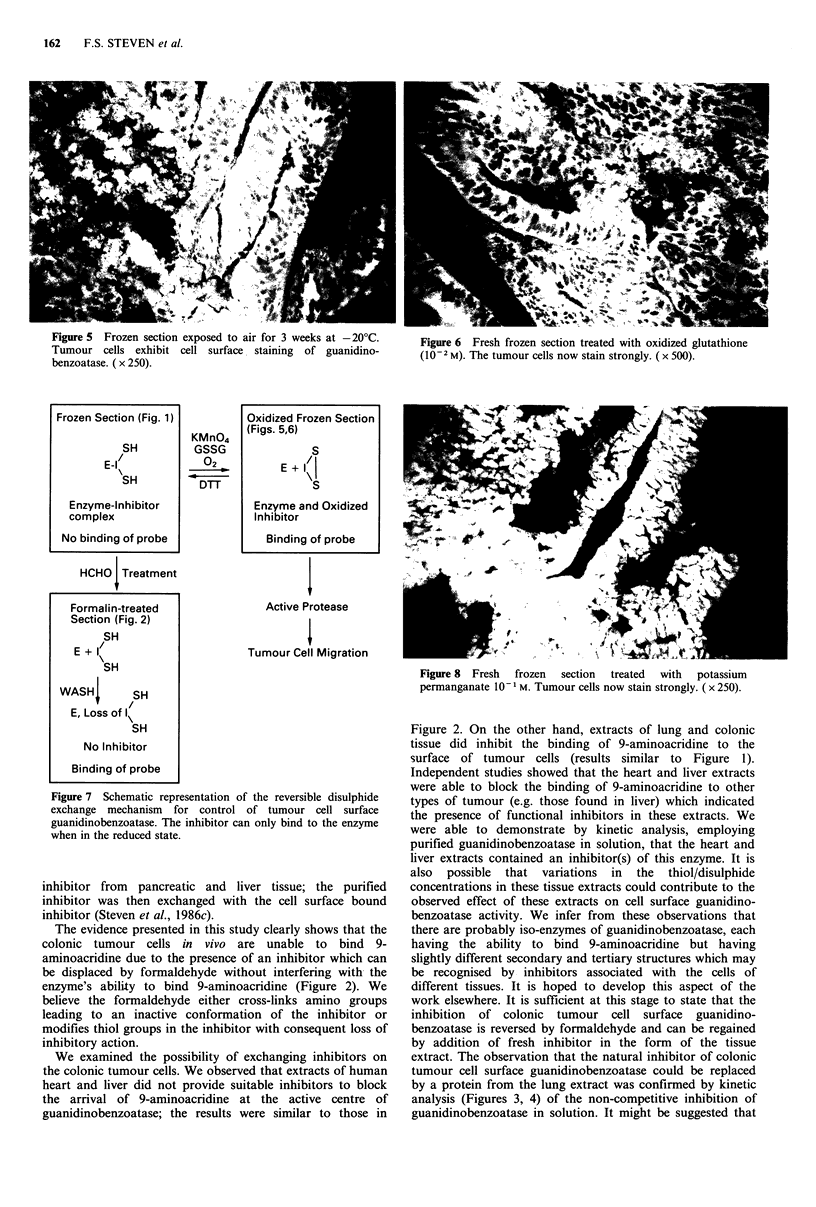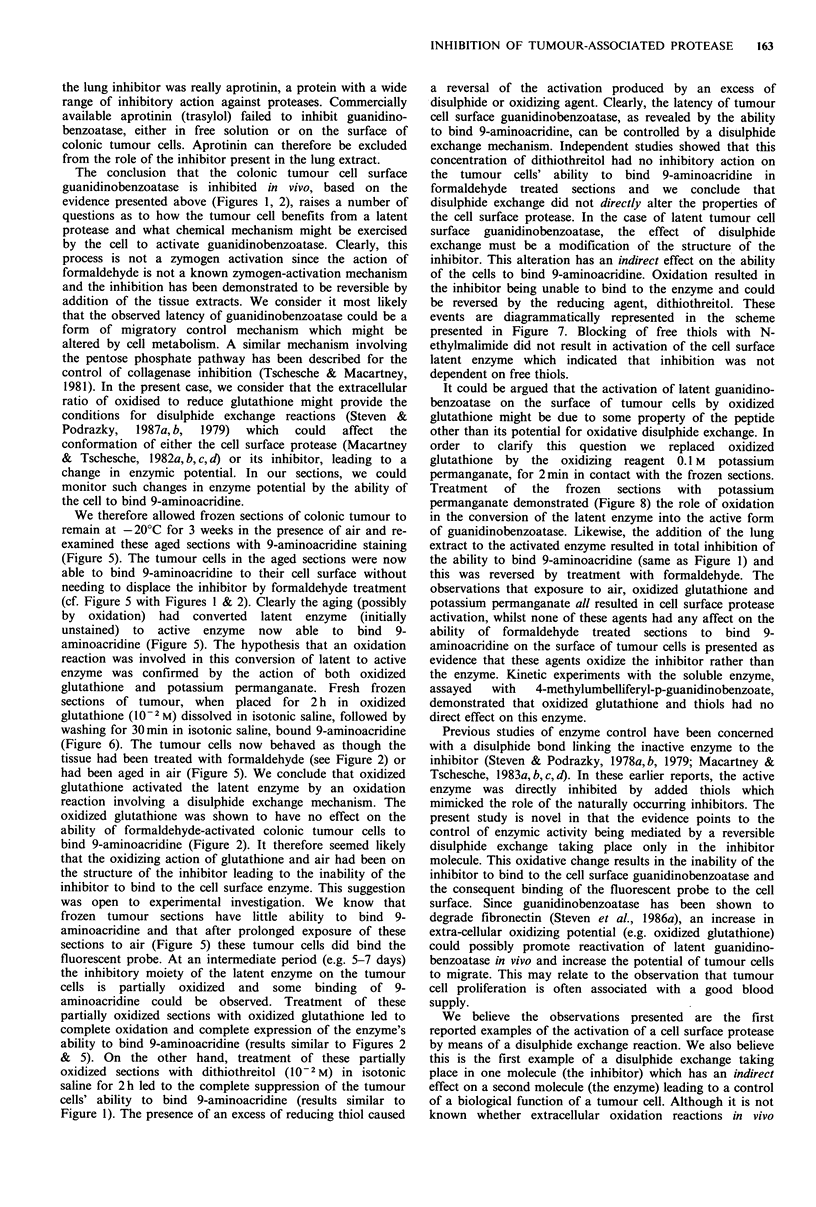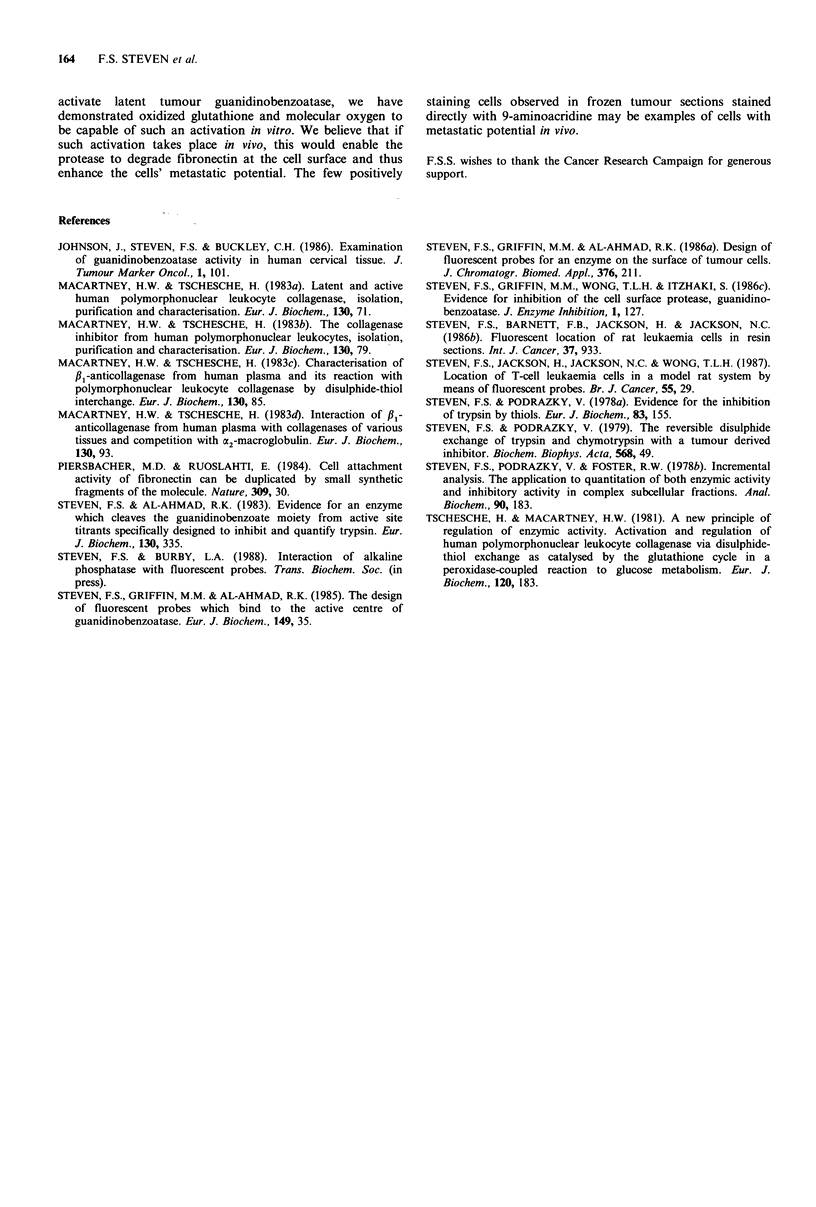# The inhibition of a tumour cell surface protease in vivo and its re-activation by oxidation.

**DOI:** 10.1038/bjc.1988.33

**Published:** 1988-02

**Authors:** F. S. Steven, H. Ali, M. M. Griffin

**Affiliations:** Department of Biochemistry and Molecular Biology, School of Biological Sciences, University of Manchester, UK.

## Abstract

**Images:**


*Br. J. Cancer* (1988), **57**, 160–164 © The Macmillan Press Ltd., 1988

### The inhibition of a tumour cell surface protease *in vivo* and its re-activation by oxidation

F.S. Steven[1], H. Ali[2] & M.M. Griffin[1]

[1]*Department of Biochemistry and Molecular Biology, School of Biological Sciences, University of Manchester, Manchester M13 9PT; and* [2]*Department of Pathology, Huddersfield Royal Infirmary, Huddersfield HD3 3EA, UK.*

**Summary** Colonic tumour cells possess a cell surface protease capable of binding 9-aminoacridine to its active centre, thus locating cells when viewed under a fluorescence microscope. *In vivo* and in frozen sections, the enzyme is masked by a protein inhibitor. This inhibitor can be displaced by formaldehyde fixation of the tissue and then replaced by adding a fresh extract of colon or lung tissue. The inhibitor is modified by oxidation; provided by air, oxidized glutathione or potassium permanganate, resulting in a change in conformation in the inhibitor and this then results in the enzyme binding the fluorescent probe. The effect of oxidation can be reversed by dithiothreitol. It is proposed that these changes are brought about by a disulphide exchange acting on the inhibitor which indirectly controls the activity of the cell surface enzyme *in vivo*.

The steps described above can be conveniently followed on sections of tissue mounted on a microscope slide; this has the advantage that the same cells can be monitored during a sequence of reactions. It is believed that these techniques could well be applied to other enzyme systems than the tumour protease described in this study.

This paper concerns a protease which is associated with cells capable of migration and in particular tumour cells (Steven *et al.*, 1985, 1986*a,b*, 1987; Johnson *et al.*, 1986). The enzyme is referred to as guanidinobenzoatase (Steven & Al-Ahmad, 1983), has been purified by affinity chromatography (Steven *et al.*, 1986*b*) and has been shown to be a protease, cleaving the arginyl bond in the peptide GlyArgGlyAsp (Steven *et al.*, 1986*c*) considered to be the link region for attachment of cells to fibronectin (Piersbacher & Ruoslahti, 1984). Previous studies have been concerned with the kinetics of inhibition of guanidinobenzoatase (Steven *et al.*, 1985; Steven & Al-Ahmad, 1983) in solution and the application of these data to the selection of fluorescent competitive inhibitors which may be used to locate cells possessing this enzyme (Steven *et al.*, 1985, 1986*a*) in formaldehyde fixed, wax embedded sections and resin embedded sections (Steven *et al.*, 1986*b*). We now present evidence from a study of fresh frozen sections demonstrating the presence of inhibitors of guanidinobenzoatase *in vivo* which control the activity of this cell surface enzyme. These inhibitors combine with the enzyme to form an inactive latent form of guanidinobenzoatase which is incapable of binding the fluorescent probes used to detect this enzyme. To illustrate this situation, we have chosen to employ frozen sections of human colonic tumours as an experimental system. The evidence is presented in the form of fluorescent micrographs which have been obtained after treating the sections with 9-aminoacridine, a successful probe for the active centre of guanidinobenzoatase (Steven *et al.*, 1985, 1986*a*). Tumour cells with active enzyme fluoresce yellow (Steven *et al.*, 1985, 1986*c*) whilst tumour cells with inhibited enzyme appear green-blue and do not fluoresce. The frozen tumour section thus replaces the reaction vessel in monitoring for the presence or absence of an inhibitor *in situ* on the cell surface protease. This test system has the advantage that the same cells can be repeatedly examined after a sequence of reactions involving the activation or inhibition of a defined enzyme, in this case guanidinobenzoatase. The same principle can be applied to other enzymes, for example alkaline phosphatase (Steven & Burby, 1988), using a suitable fluorescent probe. It would appear that the techniques described in this paper could be applied to cell surface receptors or to any specific molecule with an affinity for low molecular weight ligands.

The results of this study provide evidence for the presence of inhibitors of guanidinobenzoatase *in vivo* which can be modified by disulphide exchange reactions leading to reversible control of enzymic activity. In this case, the disulphide exchange has been shown to directly affect the structure of the inhibitor so that it can no longer react with the guanidinobenzoatase. Earlier studies (Steven & Podrazky, 1978*a,b*, 1979) demonstrated that disulphide exchange had a direct effect on trypsin-like enzymes. The later extensive studies on the role of disulphide exchange in collagenase inhibition confirmed and greatly extended these observations (Tschesche & Macartney, 1981; Macartney & Tschesche, 1983*a,b,c,d*).

### Materials and methods

Fresh frozen sections of human colonic tumours were obtained immediately after surgery. In all we examined 190 frozen sections obtained from colonic tissue taken from 6 patients. Material for wax embedding was fixed in 10% w/v formaldehyde in isotonic saline, embedded, sectioned and dewaxed by conventional histopathological techniques. Formaldehyde treatment of frozen sections was carried out for 18 h in 10% w/v formaldehyde in isotonic saline. Homogenates of human lung, liver, heart and colon were prepared by employing an Ultra-Turrax homogeniser on chopped tissue suspended in isotonic saline. The cell debris was removed by centrifugation and the supernatant extract ($\sim 1$ mg protein $ml^{-1}$) was used in the inhibition exchange experiments.

Oxidized glutathione, 4-methylumbelliferyl-p-guanidinobenzoate, N-ethylmaleimide, dithiothreitol, potassium permanganate, oxalic acid and 9-aminoacridine were purchased from Sigma Chemical Company, St. Louis, Mo., USA. Aprotinin (trasylol) was a generous gift from Bayer Chemical Company.

### Methods

The fluorescent staining technique employing 9-aminoacridine has been fully described (Steven *et al.*, 1985). Essentially, cells which posses guanidinobenzoatase in a form

Correspondence: F.S. Steven.
Received 19 June 1987; and in revised form, 21 April 1987.

**Figure 1** Fresh frozen section. No cells bind 9-aminoacridine due to the presence of the inhibitor. ( × 250).

**Figure 2** Formaldehyde-treated frozen section. The tumour cells exhibit yellow surface fluorescence in the absence of inhibitor. ( × 250).

**Figure 3** Inhibition of mouse guanidinobenzoatase by incremental additions of human lung extract, assayed with 4-methylumbelliferyl-p-guanidinobenzoate.

**Figure 4** Non-competitive inhibition of mouse guanidinobenzoatase, assayed with incremental additions of 4-methylumbelliferyl-p-guanidinobenzoate: (a) in the absence of inhibitor; and (b) in the presence of inhibitor (100 μl lung extract).

which can bind the 9-aminoacridine fluoresce a bright yellow on a blue background.

The displacement of the inhibitor from the latent enzyme was achieved by treatment with formaldehyde (see above) or by treatment for 2 h at 16°C with oxidized glutathione ($10^{-2}$ M), dissolved in isotonic saline, followed by 30 min washing in isotonic saline. Treatment with dithiothreitol ($10^{-2}$ M) dissolved in isotonic saline was for 2 h followed by a 2 min wash in isotonic saline. Alternatively, oxidation was carried out by placing the slides in 0.1 M potassium permanganate for 2 min followed by bleaching in 1% aqueous oxalic acid for 2 min and raising the pH to 7.4 with isotonic saline containing 0.1 M $NaHCO_3$ for 2 min. The replacement of inhibitor was achieved by covering the section on the slide with tissue extract and leaving for 18 h at 16°C in a wet box, followed by washing off the excess tissue extract with isotonic saline. After each of these reactions the sections were monitored by staining with 9-aminoacridine.

Simple kinetic experiments were designed to demonstrate inhibition of mouse guanidinobenzoatase (Steven & Al-Ahmad, 1983) as previously described (Steven *et al*., 1986*a*) employing 4-methylumbelliferyl-p-guanidinobenzoate as substrate. The object of these experiments was to define whether the inhibition was competitive or non-competitive. In the plot presented in Figure 4 the change in fluorescence due to the production of methylumbelliferone was used rather than the molarity of the product in the values of (l/v).

### Results and discussion

Very few fluorescent cells were observed (Figure 1) when fresh frozen sections of colonic tumour were stained directly with 9-aminoacridine and examined by fluorescent microscopy. Yet, when the *same* or identical sections were pretreated with formaldehyde prior to the staining with 9-aminoacridine, the tumour cells were clearly stained (Figure 2). Similar results to those shown in Figure 2 were obtained when formaldehyde-fixed tissues in wax embedded sections were stained with 9-aminoacridine. It was previously shown that a similar situation was found in pancreatic acinar cells (Steven *et al*., 1986*c*) and it was demonstrated that formaldehyde displaced an inhibitor from the guanidinobenzoatase. Affinity systems were used to isolate this protein

**Figure 5** Frozen section exposed to air for 3 weeks at −20°C. Tumour cells exhibit cell surface staining of guanidinobenzoatase. (×250).

**Figure 6** Fresh frozen section treated with oxidized glutathione ($10^{-2}$ M). The tumour cells now stain strongly. (×500).

**Figure 7** Schematic representation of the reversible disulphide exchange mechanism for control of tumour cell surface guanidinobenzoatase. The inhibitor can only bind to the enzyme when in the reduced state.

inhibitor from pancreatic and liver tissue; the purified inhibitor was then exchanged with the cell surface bound inhibitor (Steven *et al.*, 1986*c*).

The evidence presented in this study clearly shows that the colonic tumour cells *in vivo* are unable to bind 9-aminoacridine due to the presence of an inhibitor which can be displaced by formaldehyde without interfering with the enzyme's ability to bind 9-aminoacridine (Figure 2). We believe the formaldehyde either cross-links amino groups leading to an inactive conformation of the inhibitor or modifies thiol groups in the inhibitor with consequent loss of inhibitory action.

We examined the possibility of exchanging inhibitors on the colonic tumour cells. We observed that extracts of human heart and liver did not provide suitable inhibitors to block the arrival of 9-aminoacridine at the active centre of guanidinobenzoatase; the results were similar to those in

**Figure 8** Fresh frozen section treated with potassium permanganate $10^{-1}$ M. Tumour cells now stain strongly. (×250).

Figure 2. On the other hand, extracts of lung and colonic tissue did inhibit the binding of 9-aminoacridine to the surface of tumour cells (results similar to Figure 1). Independent studies showed that the heart and liver extracts were able to block the binding of 9-aminoacridine to other types of tumour (e.g. those found in liver) which indicated the presence of functional inhibitors in these extracts. We were able to demonstrate by kinetic analysis, employing purified guanidinobenzoatase in solution, that the heart and liver extracts contained an inhibitor(s) of this enzyme. It is also possible that variations in the thiol/disulphide concentrations in these tissue extracts could contribute to the observed effect of these extracts on cell surface guanidinobenzoatase activity. We infer from these observations that there are probably iso-enzymes of guanidinobenzoatase, each having the ability to bind 9-aminoacridine but having slightly different secondary and tertiary structures which may be recognised by inhibitors associated with the cells of different tissues. It is hoped to develop this aspect of the work elsewhere. It is sufficient at this stage to state that the inhibition of colonic tumour cell surface guanidinobenzoatase is reversed by formaldehyde and can be regained by addition of fresh inhibitor in the form of the tissue extract. The observation that the natural inhibitor of colonic tumour cell surface guanidinobenzoatase could be replaced by a protein from the lung extract was confirmed by kinetic analysis (Figures 3, 4) of the non-competitive inhibition of guanidinobenzoatase in solution. It might be suggested that the lung inhibitor was really aprotinin, a protein with a wide range of inhibitory action against proteases. Commercially available aprotinin (trasylol) failed to inhibit guanidinobenzoatase, either in free solution or on the surface of colonic tumour cells. Aprotinin can therefore be excluded from the role of the inhibitor present in the lung extract.

The conclusion that the colonic tumour cell surface guanidinobenzoatase is inhibited *in vivo*, based on the evidence presented above (Figures 1, 2), raises a number of questions as to how the tumour cell benefits from a latent protease and what chemical mechanism might be exercised by the cell to activate guanidinobenzoatase. Clearly, this process is not a zymogen activation since the action of formaldehyde is not a known zymogen-activation mechanism and the inhibition has been demonstrated to be reversible by addition of the tissue extracts. We consider it most likely that the observed latency of guanidinobenzoatase could be a form of migratory control mechanism which might be altered by cell metabolism. A similar mechanism involving the pentose phosphate pathway has been described for the control of collagenase inhibition (Tschesche & Macartney, 1981). In the present case, we consider that the extracellular ratio of oxidised to reduce glutathione might provide the conditions for disulphide exchange reactions (Steven & Podrazky, 1987*a*,*b*, 1979) which could affect the conformation of either the cell surface protease (Macartney & Tschesche, 1982*a*,*b*,*c*,*d*) or its inhibitor, leading to a change in enzymic potential. In our sections, we could monitor such changes in enzyme potential by the ability of the cell to bind 9-aminoacridine.

We therefore allowed frozen sections of colonic tumour to remain at −20°C for 3 weeks in the presence of air and re-examined these aged sections with 9-aminoacridine staining (Figure 5). The tumour cells in the aged sections were now able to bind 9-aminoacridine to their cell surface without needing to displace the inhibitor by formaldehyde treatment (cf. Figure 5 with Figures 1 & 2). Clearly the aging (possibly by oxidation) had converted latent enzyme (initially unstained) to active enzyme now able to bind 9-aminoacridine (Figure 5). The hypothesis that an oxidation reaction was involved in this conversion of latent to active enzyme was confirmed by the action of both oxidized glutathione and potassium permanganate. Fresh frozen sections of tumour, when placed for 2h in oxidized glutathione ($10^{-2}$ M) dissolved in isotonic saline, followed by washing for 30min in isotonic saline, bound 9-aminoacridine (Figure 6). The tumour cells now behaved as though the tissue had been treated with formaldehyde (see Figure 2) or had been aged in air (Figure 5). We conclude that oxidized glutathione activated the latent enzyme by an oxidation reaction involving a disulphide exchange mechanism. The oxidized glutathione was shown to have no effect on the ability of formaldehyde-activated colonic tumour cells to bind 9-aminoacridine (Figure 2). It therefore seemed likely that the oxidizing action of glutathione and air had been on the structure of the inhibitor leading to the inability of the inhibitor to bind to the cell surface enzyme. This suggestion was open to experimental investigation. We know that frozen tumour sections have little ability to bind 9-aminoacridine and that after prolonged exposure of these sections to air (Figure 5) these tumour cells did bind the fluorescent probe. At an intermediate period (e.g. 5–7 days) the inhibitory moiety of the latent enzyme on the tumour cells is partially oxidized and some binding of 9-aminoacridine could be observed. Treatment of these partially oxidized sections with oxidized glutathione led to complete oxidation and complete expression of the enzyme's ability to bind 9-aminoacridine (results similar to Figures 2 & 5). On the other hand, treatment of these partially oxidized sections with dithiothreitol ($10^{-2}$ M) in isotonic saline for 2h led to the complete suppression of the tumour cells' ability to bind 9-aminoacridine (results similar to Figure 1). The presence of an excess of reducing thiol caused a reversal of the activation produced by an excess of disulphide or oxidizing agent. Clearly, the latency of tumour cell surface guanidinobenzoatase, as revealed by the ability to bind 9-aminoacridine, can be controlled by a disulphide exchange mechanism. Independent studies showed that this concentration of dithiothreitol had no inhibitory action on the tumour cells' ability to bind 9-aminoacridine in formaldehyde treated sections and we conclude that disulphide exchange did not *directly* alter the properties of the cell surface protease. In the case of latent tumour cell surface guanidinobenzoatase, the effect of disulphide exchange must be a modification of the structure of the inhibitor. This alteration has an *indirect* effect on the ability of the cells to bind 9-aminoacridine. Oxidation resulted in the inhibitor being unable to bind to the enzyme and could be reversed by the reducing agent, dithiothreitol. These events are diagrammatically represented in the scheme presented in Figure 7. Blocking of free thiols with N-ethylmalimide did not result in activation of the cell surface latent enzyme which indicated that inhibition was not dependent on free thiols.

It could be argued that the activation of latent guanidinobenzoatase on the surface of tumour cells by oxidized glutathione might be due to some property of the peptide other than its potential for oxidative disulphide exchange. In order to clarify this question we replaced oxidized glutathione by the oxidizing reagent 0.1 M potassium permanganate, for 2min in contact with the frozen sections. Treatment of the frozen sections with potassium permanganate demonstrated (Figure 8) the role of oxidation in the conversion of the latent enzyme into the active form of guanidinobenzoatase. Likewise, the addition of the lung extract to the activated enzyme resulted in total inhibition of the ability to bind 9-aminoacridine (same as Figure 1) and this was reversed by treatment with formaldehyde. The observations that exposure to air, oxidized glutathione and potassium permanganate *all* resulted in cell surface protease activation, whilst none of these agents had any affect on the ability of formaldehyde treated sections to bind 9-aminoacridine on the surface of tumour cells is presented as evidence that these agents oxidize the inhibitor rather than the enzyme. Kinetic experiments with the soluble enzyme, assayed with 4-methylumbelliferyl-p-guanidinobenzoate, demonstrated that oxidized glutathione and thiols had no direct effect on this enzyme.

Previous studies of enzyme control have been concerned with a disulphide bond linking the inactive enzyme to the inhibitor (Steven & Podrazky, 1978*a*,*b*, 1979; Macartney & Tschesche, 1983*a*,*b*,*c*,*d*). In these earlier reports, the active enzyme was directly inhibited by added thiols which mimicked the role of the naturally occurring inhibitors. The present study is novel in that the evidence points to the control of enzymic activity being mediated by a reversible disulphide exchange taking place only in the inhibitor molecule. This oxidative change results in the inability of the inhibitor to bind to the cell surface guanidinobenzoatase and the consequent binding of the fluorescent probe to the cell surface. Since guanidinobenzoatase has been shown to degrade fibronectin (Steven *et al.*, 1986*a*), an increase in extra-cellular oxidizing potential (e.g. oxidized glutathione) could possibly promote reactivation of latent guanidinobenzoatase *in vivo* and increase the potential of tumour cells to migrate. This may relate to the observation that tumour cell proliferation is often associated with a good blood supply.

We believe the observations presented are the first reported examples of the activation of a cell surface protease by means of a disulphide exchange reaction. We also believe this is the first example of a disulphide exchange taking place in one molecule (the inhibitor) which has an *indirect* effect on a second molecule (the enzyme) leading to a control of a biological function of a tumour cell. Although it is not known whether extracellular oxidation reactions *in vivo*

activate latent tumour guanidinobenzoatase, we have demonstrated oxidized glutathione and molecular oxygen to be capable of such an activation *in vitro*. We believe that if such activation takes place *in vivo*, this would enable the protease to degrade fibronectin at the cell surface and thus enhance the cells' metastatic potential. The few positively staining cells observed in frozen tumour sections stained directly with 9-aminoacridine may be examples of cells with metastatic potential *in vivo*.

F.S.S. wishes to thank the Cancer Research Campaign for generous support.

**References**

JOHNSON, J., STEVEN, F.S. & BUCKLEY, C.H. (1986). Examination of guanidinobenzoatase activity in human cervical tissue. *J. Tumour Marker Oncol.*, **1**, 101.

MACARTNEY, H.W. & TSCHESCHE, H. (1983*a*). Latent and active human polymorphonuclear leukocyte collagenase, isolation, purification and characterisation. *Eur. J. Biochem.*, **130**, 71.

MACARTNEY, H.W. & TSCHESCHE, H. (1983*b*). The collagenase inhibitor from human polymorphonuclear leukocytes, isolation, purification and characterisation. *Eur. J. Biochem.*, **130**, 79.

MACARTNEY, H.W. & TSCHESCHE, H. (1983*c*). Characterisation of $\beta_1$-anticollagenase from human plasma and its reaction with polymorphonuclear leukocyte collagenase by disulphide-thiol interchange. *Eur. J. Biochem.*, **130**, 85.

MACARTNEY, H.W. & TSCHESCHE, H. (1983*d*). Interaction of $\beta_1$-anticollagenase from human plasma with collagenases of various tissues and competition with $\alpha_2$-macroglobulin. *Eur. J. Biochem.*, **130**, 93.

PIERSBACHER, M.D. & RUOSLAHTI, E. (1984). Cell attachment activity of fibronectin can be duplicated by small synthetic fragments of the molecule. *Nature*, **309**, 30.

STEVEN, F.S. & AL-AHMAD, R.K. (1983). Evidence for an enzyme which cleaves the guanidinobenzoate moiety from active site titrants specifically designed to inhibit and quantify trypsin. *Eur. J. Biochem.*, **130**, 335.

STEVEN, F.S. & BURBY, L.A. (1988). Interaction of alkaline phosphatase with fluorescent probes. *Trans. Biochem. Soc.* (in press).

STEVEN, F.S., GRIFFIN, M.M. & AL-AHMAD, R.K. (1985). The design of fluorescent probes which bind to the active centre of guanidinobenzoatase. *Eur. J. Biochem.*, **149**, 35.

STEVEN, F.S., GRIFFIN, M.M. & AL-AHMAD, R.K. (1986*a*). Design of fluorescent probes for an enzyme on the surface of tumour cells. *J. Chromatogr. Biomed. Appl.*, **376**, 211.

STEVEN, F.S., GRIFFIN, M.M., WONG, T.L.H. & ITZHAKI, S. (1986*c*). Evidence for inhibition of the cell surface protease, guanidinobenzoatase. *J. Enzyme Inhibition*, **1**, 127.

STEVEN, F.S., BARNETT, F.B., JACKSON, H. & JACKSON, N.C. (1986*b*). Fluorescent location of rat leukaemia cells in resin sections. *Int. J. Cancer*, **37**, 933.

STEVEN, F.S., JACKSON, H., JACKSON, N.C. & WONG, T.L.H. (1987). Location of T-cell leukaemia cells in a model rat system by means of fluorescent probes. *Br. J. Cancer*, **55**, 29.

STEVEN, F.S. & PODRAZKY, V. (1978*a*). Evidence for the inhibition of trypsin by thiols. *Eur. J. Biochem.*, **83**, 155.

STEVEN, F.S. & PODRAZKY, V. (1979). The reversible disulphide exchange of trypsin and chymotrypsin with a tumour derived inhibitor. *Biochem. Biophys. Acta*, **568**, 49.

STEVEN, F.S., PODRAZKY, V. & FOSTER, R.W. (1978*b*). Incremental analysis. The application to quantitation of both enzymic activity and inhibitory activity in complex subcellular fractions. *Anal. Biochem.*, **90**, 183.

TSCHESCHE, H. & MACARTNEY, H.W. (1981). A new principle of regulation of enzymic activity. Activation and regulation of human polymorphonuclear leukocyte collagenase via disulphide-thiol exchange as catalysed by the glutathione cycle in a peroxidase-coupled reaction to glucose metabolism. *Eur. J. Biochem.*, **120**, 183.